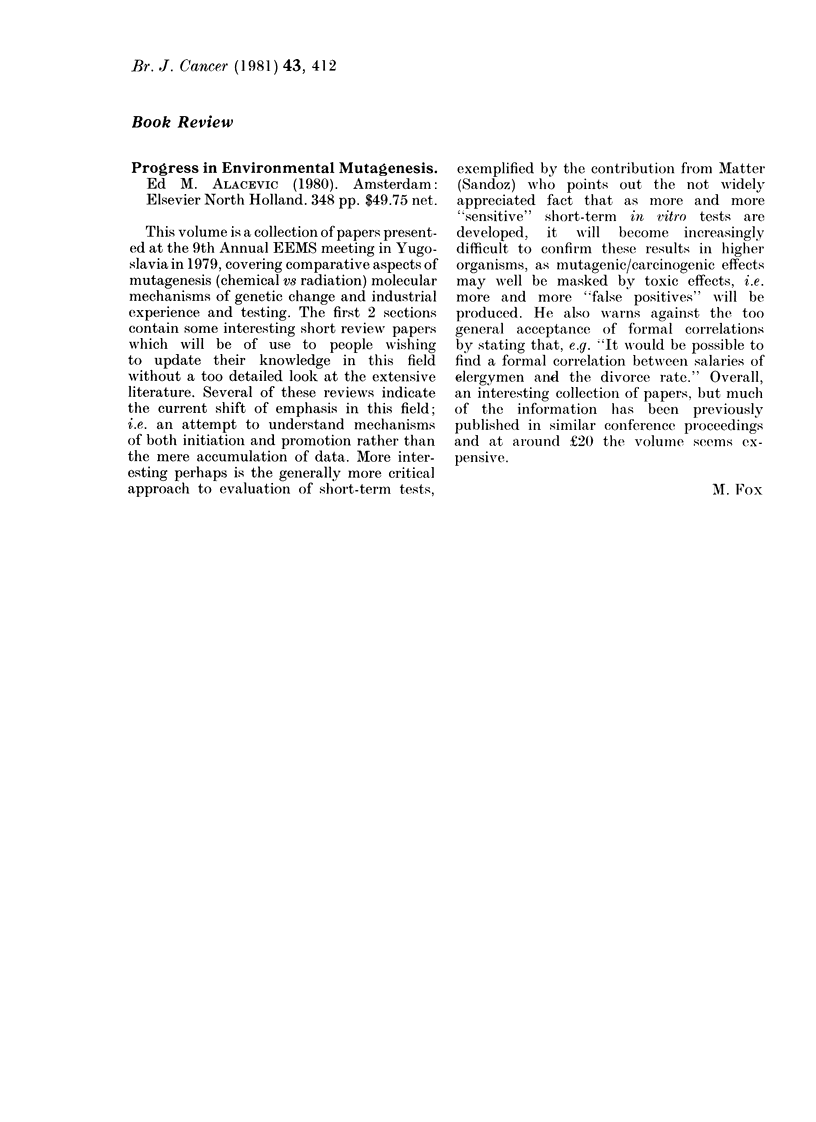# Progress in Environmental Mutagenesis

**Published:** 1981-03

**Authors:** M. Fox


### *Book Review*

**Progress in Environmental Mutagenesis.** Ed M. ALACEVIC (1980). Amsterdam: Elsevier North Holland. 348 pp. $49.75 net.

This volume is a collection of papers presented at the 9th Annual EEMS meeting in Yugoslavia in 1979, covering comparative aspects of mutagenesis (chemical *vs* radiation) molecular mechanisms of genetic change and industrial experience and testing. The first 2 sections contain some interesting short review papers which will be of use to people wishing to update their knowledge in this field without a too detailed look at the extensive literature. Several of these reviews indicate the current shift of emphasis in this field; *i.e.* an attempt to understand mechanisms of both initiation and promotion rather than the mere accumulation of data. More interesting perhaps is the generally more critical approach to evaluation of short-term tests, exemplified by the contribution from Matter (Sandoz) who points out the not widely appreciated fact that as more and more "sensitive" short-term *in vitro* tests are developed, it will become increasingly difficult to confirm these results in higher organisms, as mutagenic/carcinogenic effects may well be masked by toxic effects, *i.e.* more and more "false positives" will be produced. He also warns against the too general acceptance of formal correlations by stating that, *e.g.* "It would be possible to find a formal correlation between salaries of clergymen and the divorce rate." Overall, an interesting collection of papers, but much of the information has been previously published in similar conference proceedings and at around £20 the volume seems expensive.

M. FOX